# Outcome of Allogeneic Hematopoietic Stem Cell Transplantation in Adult Patients with Acute Lymphoblastic Leukemia: Results of a Single-Center Study

**DOI:** 10.3390/hematolrep16040062

**Published:** 2024-10-17

**Authors:** Davide Stella, Jessica Gill, Roberto Passera, Sofia Zompi, Chiara Maria Dellacasa, Ernesta Audisio, Marco Cerrano, Irene Dogliotti, Michele Dicataldo, Carolina Secreto, Benedetto Bruno, Roberto Freilone, Alessandro Busca, Luisa Giaccone

**Affiliations:** 1 Department of Molecular Biotechnology and Health Sciences, Division of Hematology, University of Torino, 10126 Torino, Italy; davide.stella@unito.it (D.S.);; 2 Stem Cell Transplant Center, A.O.U. Città della Salute e della Scienza di Torino, 10126 Torino, Italymichele.dicataldo@unito.it (M.D.);; 3 Senior Biostatistician, Department of Medical Sciences, A.O.U. Città della Salute e della Scienza di Torino, University of Torino, 10126 Torino, Italy; 4 Department of Oncology, Division of Hematology, A.O.U. Città della Salute e della Scienza di Torino, 10126 Torino, Italy

**Keywords:** acute lymphoblastic leukemia, allogeneic stem cell transplantation, total body irradiation

## Abstract

Background: Despite the adoption of pediatric-like chemotherapy protocols, the introduction of new immunotherapies and a better understanding of the oncogenic landscape, the outcome for adult patients with acute lymphoblastic leukemia (ALL) remain substantially dismal. The aim of the present study was to evaluate the outcome in terms of survival in a cohort of adult patients with ALL who received allogeneic hematopoietic stem cell transplantation (alloSCT) between 2013 and 2023. Methods: This was a single-center observational retrospective study including all consecutive adult patients with ALL who received an alloSCT between April 2013 and April 2023 at the Stem Cell Transplant Center AOU Città della Salute e della Scienza of Torino. The primary endpoints were overall survival (OS), graft-versus-host disease (GVHD) Relapse-Free Survival (GRFS), Leukemia-Free Survival (LFS) and cumulative incidence (CI) of Non-Relapse Mortality (NRM). Results: The 4-year OS and LFS were 63.4% and 48.1%, respectively, and the 1-year GRFS was 42.9%. The 1-year CI of bloodstream infections (BSI), invasive fungal infections and NRM were 38%, 7% and 18.4%, respectively. Multivariate analysis showed that the use of total body irradiation (TBI), a time interval from diagnosis to alloSCT 7 months and female gender were factors significantly associated with better OS. Relapse of the underlying malignancy and BSI were the main causes of death. Conclusion: Our study suggests that alloSCT from a matched sibling donor (MSD) and alternative donors may be considered an effective tool for patients with ALL achieving a CR.

## 1. Introduction

The widespread use of pediatric-inspired protocols and the curative potential of immunotherapeutic strategies, in parallel with deeper insights into the molecular biology of acute lymphoblastic leukemia (ALL), have had a remarkable impact on the outcome of adult ALL [1].

While the majority of children and adolescents with newly diagnosed ALL are nowadays cured, with overall survival (OS) reaching approximately 80% [2,3], the outcome for adults, particularly those aged over 55 years, continues to be poor, with long-term remission rates of only 30–40% [4,5,6].

The role of allogeneic hematopoietic stem cell transplantation (alloSCT) in patients with Ph-negative ALL is an area of active investigation, in particular for patients in complete remission (CR1). A Cochrane systematic review and meta-analysis [7] of 14 controlled trials, including adult ALL in CR1, showed that disease-free survival (DFS) and OS were significantly better for the donor arms versus the non-donor arms, and similar results have been reported in other studies [8,9,10,11,12].

The definition of disease risk has also evolved over time, with post-treatment measurable residual disease (MRD) being considered the most important prognostic factor driving alloSCT indication as opposed to only clinical characteristics at diagnosis [13,14]. There has been a surge of evidence suggesting that patients with standard-risk ALL who achieve MRD-negative CR1 may achieve outcomes roughly superimposable with continued chemotherapy alone without upfront alloSCT [15,16].

Indeed, the majority of published studies evaluated the results of alloSCT using different conditioning regimens and different donors [17,18,19], while scant studies assessed overall transplant outcomes [20]. The use of grafts from haploidentical and unrelated donors has broadened the application of alloSCT to a large number of patients who might benefit from the procedure; however, mortality associated with transplant-related complications, occurring in 10 to 20% of patients, may be considered the main limiting factor for the successful outcome of patients with ALL receiving alloSCT.

Despite OS in adults not changing a lot over the past 10 years, many improvements in allograft management have occurred. Therefore, in this single-center study, we aimed to analyze the results of alloSCT in adult patients with ALL and included a detailed evaluation of infectious complications and graft-versus-host disease (GVHD) to identify prognostic factors potentially affecting the outcome.

## 2. Patients and Methods

### 2.1. Study Design and Data Collection

This was a single-center observational retrospective study including all consecutive adult patients with ALL who received an alloSCT between April 2013 and April 2023 at the Stem Cell Transplant Center AOU Città della Salute e della Scienza of Torino.

Patients receiving multiple alloSCTs during the study period were censored at the time of the second alloSCT and data were collected independently for each single transplant.

Indications for alloSCT were high-risk ALL (age at diagnosis > 60 years, high white blood cell count at diagnosis, complex karyotype, the presence of an immature cell phenotype) or relapsing ALL or MRD-positivity at a certain timepoint during chemotherapy.

Written consent for transplant procedures and for the use of patients’ medical records for research purposes was obtained from all subjects involved in the study.

Data collected included donor and recipient characteristics (age, gender, cytomegalovirus [CMV] serostatus, Hematopoietic Cell Transplantation-specific Comorbidity Index [HCT-CI or SORROR score]), disease features (molecular subtypes, disease status at transplant, previous therapies) and transplant details (year of transplant, type of conditioning regimen, stem cell source, and GVHD prophylaxis regimen).

Patients received a myeloablative conditioning regimen (MAC) or reduced-intensity conditioning (RIC) according to their clinical status and HCT-CI.

MAC contained total body irradiation (TBI) with a dose > 6 Gy, or a total dose of intravenous busulfan (Bu) > 6.4 mg/kg or a cyclophosphamide dose > 120 mg/kg (or >60 mg/kg if in combination with other drugs).

Per institutional policy, patients over 55 years of age or with significant pre-transplant comorbidities (SORROR Score ≥ 3) preferentially received RIC regimens with thiotepa, busulfan and fludarabine.

Regimens for GVHD prophylaxis were as per institutional protocols. GVHD prophylaxis regimens included cyclosporin (CSA) and short-course methotrexate (MTX) or CSA combined with mycophenolate mofetil (MMF). In vivo T-cell depletion with thymoglobulin (ATG) was used in transplants from unrelated donors, mainly as 5–7 mg/kg divided into two or three doses. Patients transplanted from haploidentical donors received tacrolimus, MMF and post-transplant cyclophosphamide (PTCY), as described by Luznik et al. [21].

Due to the retrospective observational nature of this trial and according to Italian law (Agenzia Italiana del Farmaco-AIFA, Guidelines for observational studies, 20 March 2008), no formal approval from the local Institutional Review Board/Independent Ethics Committee was needed.

### 2.2. Transplant Procedure and Supportive Care

Candidates for allograft were cared for in single rooms with positive pressure and high-efficiency particulate air filter (HEPA) filters at least until engraftment. All patients had an indwelling bilumen central line catheter (CVC) placed at the time of admission, and nursing staff guaranteed periodical dressing and site care. CVC was maintained for the first 3 months after stem cell infusion if infection, venous thrombosis or additional complications did not occur.

Granulocyte-colony stimulating factor (G-CSF) was not routinely administered after alloSCT, except for patients grafted from haploidentical donors.

Until 2017, fluoroquinolones (FQ) prophylaxis with levofloxacin 500 mg was given to all patients daily via oral administration, from day +1 until neutrophil engraftment; after 2017, no universal antibacterial prophylaxis was given to patients receiving alloSCT.

Fluconazole was used as an antifungal prophylaxis from day 0 until day +75 in matched sibling donor (MSD) and matched unrelated donor (URD) transplants; Posaconazole was used if GVHD occurred. Until 2020, haploidentical alloSCT recipients received micafungin (50 mg/day) from day 0 to neutrophil engraftment, followed by fluconazole until day +75; from 2020 onward, all haploidentical alloSCT recipients received fluconazole until day +75.

For the first post-alloSCT year, acyclovir (400 mg given twice daily) and cotrimoxazole (given 3 times per week) were provided as anti-herpes simplex virus and anti-pneumocystis and toxoplasma prophylaxis, respectively.

Since 2019, Letermovir (LTV) has been used as a CMV reactivation prophylaxis from day +7 until day +100 in patients seropositive for CMV who are receiving [R+] transplants. Patients received LTV at a dosage of 480 mg once daily. In the case of co-administration with cyclosporine, we reduced the dosage of LTV to 240 mg once daily.

During hospitalization after alloSCT, the following diagnostic approach was used if body temperature > 38 °C was detected: initiation of broad-spectrum empiric antibiotic therapy, unless there was isolation of a pathogen sensitive to other antibiotics; performance of at least two sets of blood cultures on peripheral venous catheter and central venous catheter; and screening for invasive aspergillosis using serum galactomannan antigen testing. A lung high-resolution computed tomography (HRCT) was performed on the third day of persisting fever and/or in case of respiratory symptoms.

Viremia for CMV and Epstein–Barr Virus (EBV) were assessed pre-alloSCT and then biweekly for CMV-DNA and weekly for EBV-DNA. Additional detection of circulating viral copies of CMV-DNA and EBV-DNA was carried out in case of suspected disease caused by reactivation of the aforementioned viruses.

In addition, pre-alloSCT serology of CMV (IgG and IgM) and EBV (VCA IgG and VCA IgM) was evaluated.

### 2.3. Definitions

ALL was classified according to the World Health Organization (WHO) classification (2022). Specifically, we divided the patients into B-ALL and T-ALL. Among B-ALL, we describe B-ALL Ph+ and B-ALL Ph− according to the presence or absence of t (9;22), respectively.

Neutrophil engraftment was defined as the first of three consecutive days with neutrophils >0.5 × 10^9^/L following alloSCT. Platelet engraftment was defined as the first of seven consecutive days with platelets >20 × 10^9^/L without transfusion.

Both acute GVHD and chronic GVHD diagnoses were assessed based on clinical symptoms and/or biopsies according to standard criteria [22]. Severity of chronic GVHD was defined according to National Institutes of Health criteria [23,24].

## 3. Statistical Analysis and Study Endpoints

The primary endpoints were OS, GVHD Relapse-Free Survival (GRFS), Leukemia-Free Survival (LFS) and cumulative incidence (CI) of Non-Relapse Mortality (NRM). OS was defined as the time from transplant to death from any cause; GRFS as the time from transplant to the first event among grade II–IV aGVHD, moderate-severe chronic GVHD, hematologic relapse and death from any cause; LFS was defined as survival with no evidence of relapse or progression. OS, GRFS and LFS curves were estimated by the Kaplan–Meier method. OS was also estimated by the Cox proportional hazard regression model. As for NRM, death without relapse was the main event, while relapse was a competing event.

The secondary endpoints were the CIs of four different infectious events: bloodstream infections (BSI) occurrence, invasive fungal infections (IFI) occurrence, CMV reactivation and EBV reactivation in the first 12 months after bone marrow transplant (main events). Relapse/death without BSI occurrence, IFI occurrence, CMV reactivation and EBV reactivation were the competing events. The CI curves were compared by the Gray test.

The following covariates were tested as potential risk factors: recipient age (over vs. under median), recipient gender (male vs. female), phenotype (T vs. B Ph− vs. B Ph+), disease status at transplant (advanced vs. CR2/CR3 vs. CR1), SORROR Comorbidity Index (≥3 vs. 0–2), donor type (haploidentical vs. URD vs. matched sibling donor [MSD]), stem cell source (peripheral blood vs. bone marrow), conditioning regimen (RIC vs. MAC), antibiotic prophylaxis (any vs. none), TBI regimen (yes vs. none), fungal prophylaxis (secondary vs. micafungin vs. fluconazole), GVHD prophylaxis (CSA-MTX vs. calcineurin inhibitor (CNI)-MMF-PTCY vs. ATG), EBV reactivation, CMV reactivation, BSI occurrence, IFI occurrence (any vs. none), acute GVHD (grade II–IV vs. none-I) occurrence, chronic GVHD (moderate/severe vs. none/mild) occurrence and year of transplant (2017–2023 vs. 2013–2016). Main patient characteristics for categorical risk factors were described as absolute/relative frequencies and compared by Fisher’s exact test; continuous covariates were reported as median/interquartile range (IQR) and compared by the Mann–Whitney test. All *p*-values were obtained by the two-sided exact method at the conventional 5% significance level. The overall follow-up was updated on 15 April 2024 and the data as of June 2024 were analyzed in R 4.3.2 (R Foundation for Statistical Computing, Vienna-A, http://www.R-project.org, accessed on 16 October 2024).

## 4. Results

### 4.1. Patients, Disease and Transplant Characteristics

Overall, 69 cases with ALL receiving an alloSCT have been included in the present study (Table 1). The median age at alloSCT was 40 years (median IQR: 29–48 years) and 49 cases (71.0%) were male. The diagnosis was Ph-negative B-ALL, Ph-positive B-ALL and T-ALL in 35 (50.7%), 10 (14.5%) and 24 (34.8%) cases, respectively. The median time from diagnosis of the underlying disease to alloSCT was 7 months (median IQR: 6–13 months). Among the ALL Ph+ patients for whom MRD data were analyzed at transplantation, seven were MRD-negative and three were MRD-positive.

Seven cases (10.1%) presented central nervous system (CNS) involvement at the time of diagnosis.

Fourteen cases underwent next-generation antibody therapy; specifically, 13 cases (13/69, 18.8%) received the bispecific antibody Blinatumomab, four (4/69, 5.8%) received the drug-conjugated antibody Inotuzumab ozogamicin, and three received both of them. In all but one of the above cases, the antibody was used after disease recurrence as a bridge to alloSCT.

Among cases treated with blinatumomab before transplant, six (46.2%) had BSI. We checked if any of these had infections before transplant and only two patients were part of this group.

Among patients with Ph + B-ALL, one was treated with ponatinib before alloSCT, and overall, three patients (4.3%) received ponatinib as maintenance therapy (including the pre-treated one).

Regarding disease status at transplant, 67 cases (97.1%) were in complete remission and two cases had progressive disease (2.9%).

Overall, 19 cases (27.5%) received alloSCT from an MSD, while 50 cases (72.5%) were grafted from alternative donors, including URD in 34 cases and haploidentical family donors in 16 cases.

Most cases received MAC (65/69, 94.2%); in detail, TBI (12 Gy over 3 days) containing regimens were used in 47 cases (68.1%). Four patients (5.8%) received RIC regimens; among these, only one patient experienced reduced TBI as a 200 Gy single dose. Busulfan-based preparative regimens were used in 17 patients (24.6%).

As expected, peripheral blood stem cells (PBSCs) were the graft source in 95.7% of the cases (66/69).

GVHD prophylaxis with CNI-MTX and ATG was used in 33 cases (47.8%), almost all URD alloSCT; 17 cases (24.6%) grafted from haploidentical donors received PTCY; CSA and MTX were the GVHD prophylaxis in 19 (27.6%) transplant recipients.

### 4.2. Clinical Outcomes

Overall, 65 cases achieved a neutrophil count greater than 500/mcl, and four patients died early after transplant (median day +10) without evidence of neutrophil recovery. The median time to engraftment was 17 days for neutrophil count (range 12–34 days) and 13 days for platelet count (range 8–41 days). No significant differences in terms of time to engraftment were observed among patients receiving transplants from MSD, URD or haploidentical donors.

**Figure 1:**
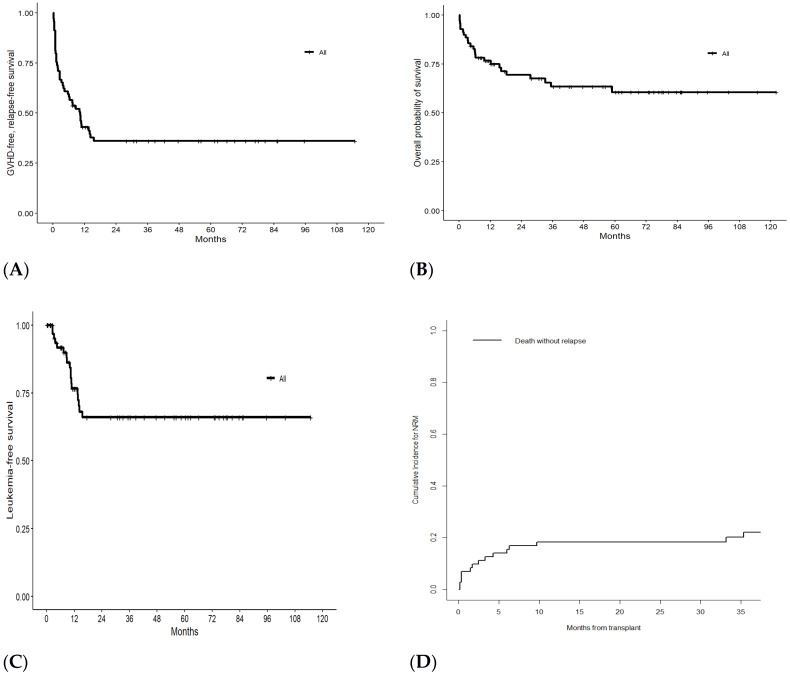
GRFS (**A**), OS (**B**), LFS (**C**) and cumulative incidence of Non-Relapse Mortality (**D**) of 69 patients with ALL who received alloSCT.

Grade II–IV aGVHD occurred in 14 cases (20.3%) with a 100-day cumulative incidence of 19.7% (Supplementary Figure S1). A total of 9 cases out of 60 (15.0%) evaluable patients had moderate to severe cGVHD, with a CI at 6 and 12 months post-alloSCT of 5.6 and 9.9%, respectively (Supplementary Figure S2). The 1-year GRFS was 41.6% (Figure 1A). The aGVHD and cGVHD incidences did not differ significantly based on the different types of transplants (MSD vs. URD vs. haplo-alloSCT).

Among 65 evaluable cases, BSIs occurred in 28 (43.1%), with a cumulative incidence at 1–3–12 months of 29.6–36.6 and 38.0%, respectively (Supplementary Figure S3). Five cases had a probable/proven IFI (7.4%), with a cumulative incidence at 1–3–12 months of 2.8–5.6 and 7.0%, respectively (Supplementary Figure S4). Clinically significant CMV reactivation was reported in 14 cases (20.6%), with a cumulative incidence at 1–3–12 months of 4.2–19.7 and 19.7%, respectively (Supplementary Figure S5); of note, only six cases in our cohort received prophylaxis with LTV and none of them had CMV reactivation. Four cases (5.9%) of EBV reactivation were reported in the first month post-alloSCT, with a cumulative incidence at 3 months of 4.3% (Supplementary Figure S6). All cases were successfully treated with Rituximab, none of them was able to discontinue immune suppression and no PTLD was observed.

Veno-occlusive disease (VOD) developed in one patient previously treated with inotuzumab. Weekly fibroscan monitoring prompted early diagnosis and treatment with Defibrotide, with subsequent complete resolution of the VOD.

The median follow-up for the whole cohort was 56 months (range IQR: 17–78 months). Overall, 45 patients were alive at the last follow-up: 13 in the MSD, 24 in the URD and eight in the haplo-alloSCT group.

The median OS was not reached: the 3- and 4-year OS was 63.4% (Figure 1B). The 3- and 4-year OS was 69.5% after excluding the second alloSCT. In detail, the survival data according to ALL subtype are as follows: six patients alive out of 10 (60%) among the ALL-Ph+, 21 out of 35 (60%) among the ALL-Ph− and 18 out of 24 (75%) among the ALL-T. Considering disease status at transplantation, the survival data are as follows: 37 patients alive out of 49 (76%) among CR1 patients, 7 out of 14 (50%) among CR2 patients and 0 out of 4 (0%) among CR3 patients (Supplementary Figure S7).

Time from diagnosis to BMT of less than 7 months, a TBI-containing regimen and female gender were significantly associated with better OS in univariate analyses (Supplementary Figure S8), while a nonsignificant trend was observed for HCT-CI ≥ 3 (*p* = 0.061) and BSI occurrence (*p* = 0.092); all the other covariates were not statistically significant (Table 2). Disease relapse occurred in 17 patients (27.5%) with an RI of 29.5% 3 and 5 years post-alloSCT. LFS at 3 and 4 years was 48.1% (Figure 1C).

The probabilities of NRM were 18.4% and 22.1% at 1 and 3 years, respectively (Figure 1D). The median time to NRM was 3 months. The 1- and 3-year NRM was 9.6% and 13.8%, respectively, after excluding patients undergoing a second alloSCT.

### 4.3. Second Transplant

Overall, six patients underwent a second alloSCT for disease recurrence post first alloSCT.

The median age at second alloSCT was 32 years (range IQR: 31–42 years). All of them were in CR at the time of transplant and received a MAC regimen.

All patients died of transplant-related complications, with a median death from transplant of 73 days (range IQR: 21–123 days) from the second transplant.

### 4.4. Causes of Death

Considering the whole cohort, 24 patients died (34.8%), six in the MSD, 10 in the URD and eight in the haplo-alloSCT cohorts. Recurrence of the underlying disease accounted for 29% of deaths (7/24) and transplant-related complications were the main cause of mortality (17/24, 71%). In this group, the causes of death were sepsis (seven patients, 41.2%), secondary malignancy (two patients, 11.8%), post-transplant microangiopathy (two patients, 11.8%), multiple organ failure (two patients, 11.8%), unknown cause (two patients, 11.8%) and suicide (one patient, 5.9%). One patient, who underwent two transplants, died of cGVHD complications.

## 5. Discussion

Adult ALL prognosis is still dismal and alloSCT is generally considered a curative treatment option for this class of patients. In the present study, we evaluated 69 adult ALL cases who received an alloSCT from MSD or alternative donors.

Our results are remarkably encouraging as they demonstrated 3-year OS, LFS and GRFS of 63.4%, 48.1% and 36.0%, respectively. These findings compare favorably with those reported by other authors.

In 2015, we reported our preliminary results in 40 patients with ALL receiving an alloSCT between 2009 and 2011: the 5-year OS and DFS were 53%, supporting some improvements in transplant management as well as the persistence of an unmet clinical need in terms of disease control [25].

Kiehl et al. [26] reported a 3-year DFS of 29% in 264 patients with ALL receiving alloSCT from MSD and URD. A large retrospective analysis by EBMT [27] reported the results of alloSCT for 2304 adults with ALL in CR1 showing a 2-year LFS rate reaching 60% for both MSD- and URD-alloSCT. Santoro et al. [28] analyzed 208 adult patients with ALL (including T-ALL in 34% of cases) who received unmanipulated haploidentical alloSCT (and PTCY in 57% of cases): 3-year OS, LFS and GRFS were 33%, 31% and 26%, while NRM was 32%. Several factors may potentially explain our findings. Overall, patients were young, with a median age of 40 years and a low burden of comorbidities, as demonstrated by the HCT-CI ≤ 2 in a consistent number of patients (70%). According to these observations, over 90% of the patients were able to tolerate a MAC, including TBI in the majority of cases (47/65, 72%). Notwithstanding these considerations, the NRM was not negligible (17.5% at 1 year), with half of the deaths due to bacterial infections, while viral infections (CMV and EBV) did not seem to have a clinical relevance. In this respect, it should be emphasized that antibacterial prophylaxis was not part of our policy, and we do not know whether stewardship including antibiotic prophylaxis might supersede this complication. In contrast, the rate of IFI was consistently low, and one patient receiving only inotuzumab before alloSCT developed VOD/SOS.

The disease status has a remarkable impact on the outcome of patients receiving alloSCT. Several lines of evidence suggest that the achievement of CR1 or CR and even more importantly a molecular CR are strong predictors of long-term survival of ALL patients who are candidates for alloSCT. Over 90% of our patients were grafted in CR (MRD+ (4), CR1 (45), CR2-CR3 (18)); regrettably, molecular analysis was not available at the time of alloSCT and only a few were treated with blinatumomab or inotuzumab before alloSCT, hindering evaluation of the potential impact of these drugs on patient outcomes.

One of the major findings of our study is that a favorable outcome for patients receiving alloSCT for the treatment of adult ALL may be achieved even with the use of alternative donors. This finding is not unprecedented, since several clinical studies have demonstrated comparable outcomes in MSD, haplo-alloSCT and MUD donors in ALL [27,29,30,31].

In total, 72% of the cases were grafted from alternative donors (10/10 HLA MUD in 27% of the cases and 9/10 mismatched URD in 21% of the cases). A triple combination of CNI-MTX and ATG (5 mg/kg over two days) was used in this setting, leading to an excellent rate of engraftment and good control of GVHD; the use of PTCY was employed in patients who received haplo-alloSCT, achieving similar results. As a result, the GRFS was 42.9%, similar to that reported by Nagler et al. [27] (49.6%) in ALL patients grafted with haploidentical donors.

More recently, the use of chimeric antigen receptor-engineered T (CAR-T) cells has revolutionized the therapeutic paradigm of patients with B-cell lymphoid malignancies, but even patients with ALL may benefit from this strategy and its role has been rapidly evolving in this setting. In this respect, incorporating CAR-T cells in the therapeutic algorithm that includes alloSCT represents an appealing option to circumvent disease progression and improve the outcomes for patients with ALL [32].

We are aware that our study has several limitations. This is a single-center study restricted to a small number of patients. Based on the retrospective nature of the study, the results should be considered suggestive rather than conclusive. Lastly, there are unmeasured factors such as missing MRD and the use of immunotherapeutic agents that have not been considered and may affect the results.

Despite the above limitations, our results confirm that alloSCT from both MSD and alternative donors remains an effective tool for patients with ALL achieving a CR. The impact of new therapies on NRM and OS remains to be defined in prospective trials including a large number of patients.

## Figures and Tables

**Table 1:** Main patients’ characteristics.

Characteristics	Overall
Number of patients	69
Sex, male/female (male %)	49/20 (71.0%)
Age at transplant (years), median (IQR)	40 (29–48)
Year of transplantation	
2013–2016	34 (49.3%)
2017–2023	35 (50.7%)
Time from diagnosis to transplant (mos), median (IQR)	7 (6–13)
Primary disease	
B Ph+	10 (14.5%)
B Ph−	35 (50.7%)
T	24 (34.8%)
Disease risk	
High risk	42 (60.9%)
Standard risk	27 (39.1%)
Disease status at transplant	
CR1	49 (71.0%)
CR2	14 (20.3%)
CR3	4 (5.8%%)
Relapse/progression	2 (2.9%)
MRD status in B Ph+	
MRD negative	7 (70.0%)
MRD positive	3 (30%)
Donor type	
MSD	19 (27.5%)
URD 10/10	19 (27.5%)
URD 9/10	15 (21.7%)
Haploidentical	16 (23.2%)
HCT-CI	
0–2	43 (69.4%)
≥3	19 (30.6%)
Stem cell source	
BM	3 (4.3%)
PBSC	66 (97.1%)
Conditioning regimen	
MAC	65 (94.2%)
TBI-based	47 (72.3%)
Busulfan-based	17 (26.2%)
Other	1 (1.5%)
RIC	4 (5.8%)
Busulfan-based	2 (50.0%)
Cy-Tt	1 (25.0%)
Cy-Flu-TBI 200	1 (25.0%)
TBI	
no	21 (30.9%)
yes	47 (69.1%)
CMV serology	
R+/D−	23 (33.3%)
Other	44 (63.8%)
unknown	2 (2.9%)
GVHD prophylaxis	
ATG	33 (47.8%)
CNI-MMF-PT/Cy	17 (25.0%)
CSA-MTX	19 (27.9%)
CD34 × 10^6^/kg, median (IQR)	6.8 (6.1–8.7)
CD3 × 10^6^/kg, median (IQR)	2.2 (1.7–3.1)

Abbreviations: IQR, interquartile range; CR, complete remission; MSD, matched related donor; URD, matched unrelated donor; HCT-CI, hematopoietic cell transplantation comorbidity index; BM, bone marrow; PBSC, peripheral blood stem cell; MAC, myeloablative conditioning; TBI, total body irradiation; RIC, reduced intensity conditioning; Cy, cyclophosphamide; Tt, thiotepa; CMV, cytomegalovirus; GVHD, graft-versus-host disease; CSA, cyclosporin; MTX, methotrexate; ATG, anti-thymocyte globulin; PT/Cy Post transplantation Cyclophosphamide; CNI calcineurin inhibitor; MMF mycophenolate mofetil; neg, negative; pos, positive.

**Table 2:** Univariate and multivariate Cox proportional hazard regression for OS.

Risk Factors	Univariate Analysis		Multivariate Analysis	
	HR (95% CI)	*p*	HR (95% CI)	*p*
Time from diagnosis to alloSCT (7+ vs. ≤7), months	2.57 (1.11–5.94)	0.027	1.55 (0.64–3.73)	0.329
Gender (male vs. female)	3.99 (1.20–13.34)	0.025	4.49 (1.33–15.21)	0.016
TBI (yes vs. no)	0.22 (0.10–0.49)	<0.001	0.20 (0.09–0.45)	<0.001

Abbreviations: HR, hazard ratio; CI, confidence interval; alloSCT, allogeneic hematopoietic stem cell transplant; vs., versus; TBI, total body irradiation.

## Data Availability

On request.

## Supplementary Materials

The following supporting information can be downloaded at: https://www.mdpi.com/article/10.3390/hematolrep16040062/s1, Figure S1. Cumulative incidence of grade II-IV aGVHD.; Figure S2. Cumulative incidence of moderate to severe cGVHD.; Figure S3. Cumulative incidence of bloodstream infections (BSI).; Figure S4. Cumulative incidence of probable/proven invasive fungal infection (IFI).; Figure S5. Cumulative incidence of clinically significant cytomegalovirus (CMV) reactivation.; Figure S6. Cumulative incidence of and Ebstein-Barr (EBV) reactivation; Figure S7. Overall survival considering disease status at transplantation; Figure S8. Overall survival: in detail TBI vs. non-TBI.
