# Supplementary material for: Outcome of Allogeneic Hematopoietic Stem Cell Transplantation in Adult Patients with Acute Lymphoblastic Leukemia: Results of a Single-Center Study
Source: Hematol Rep. 2024 Oct 17;16(4):636–47. doi: 10.3390/hematolrep16040062 (PMC11503301; doi:10.3390/hematolrep16040062)
Supplement: Supplementary file 1 [file hematolrep-16-00062-s001.zip › hematolrep-3170628-supplementary.pdf]

Supplementary

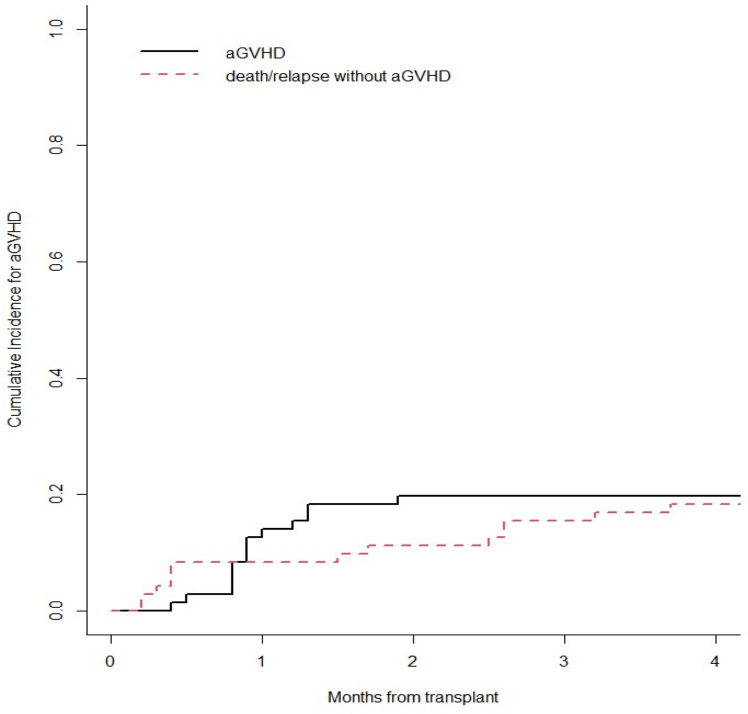

Figure S1. Cumulative incidence of grade II-IV aGVHD.

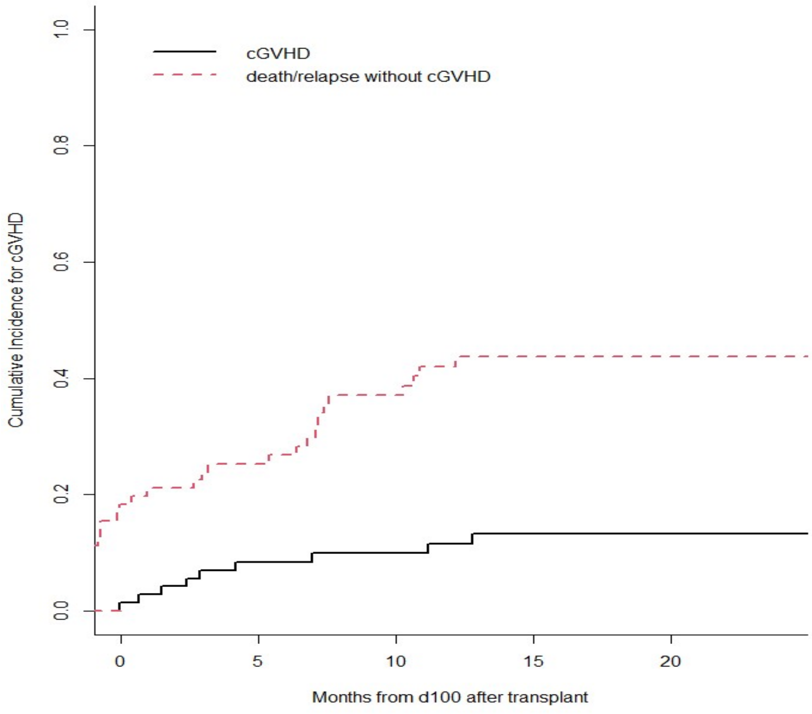

Figure S2. Cumulative incidence of moderate to severe cGVHD.

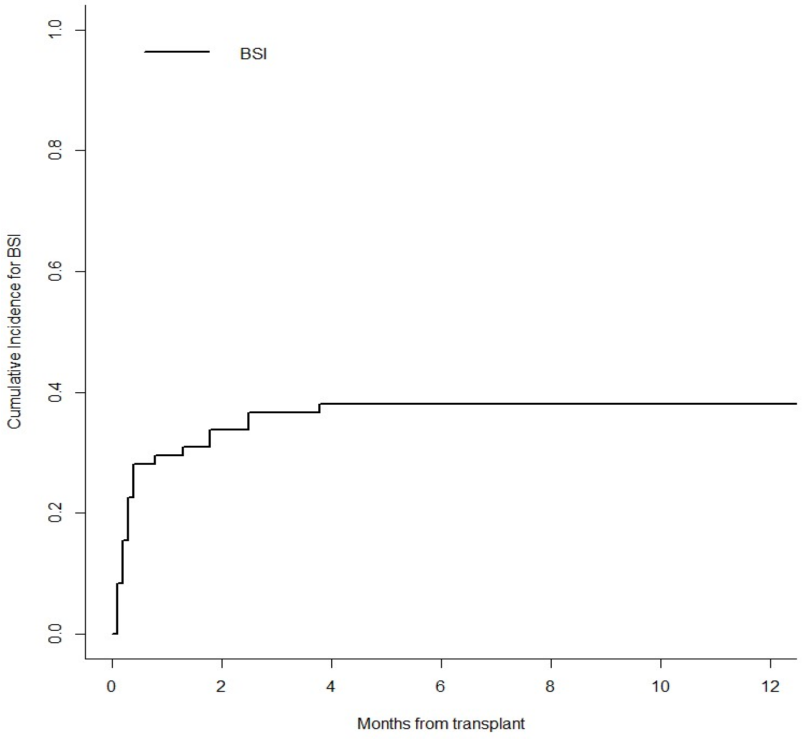

Figure S3. Cumulative incidence of bloodstream infections (BSI).

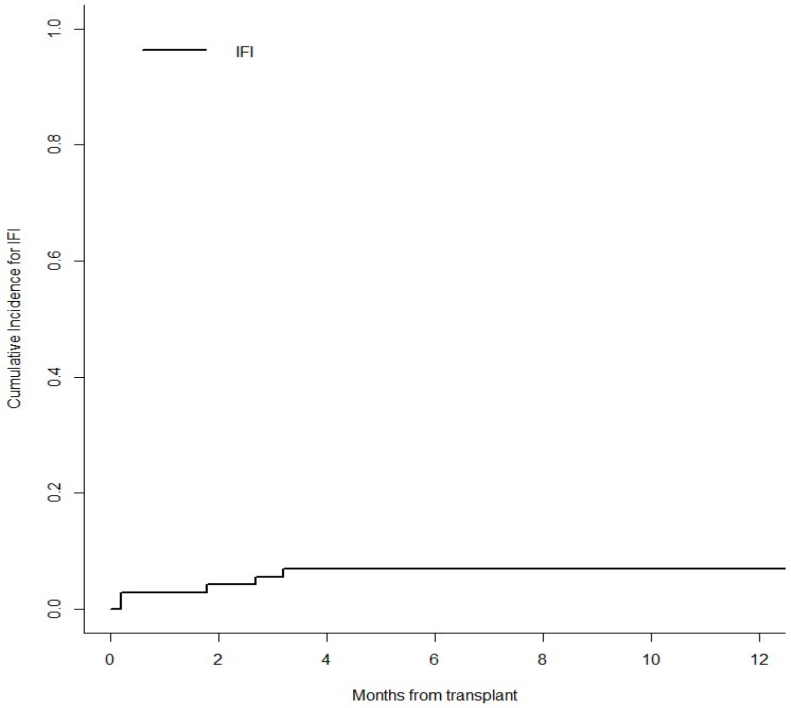

Figure S4. Cumulative incidence of probable/proven invasive fungal infection (IFI).

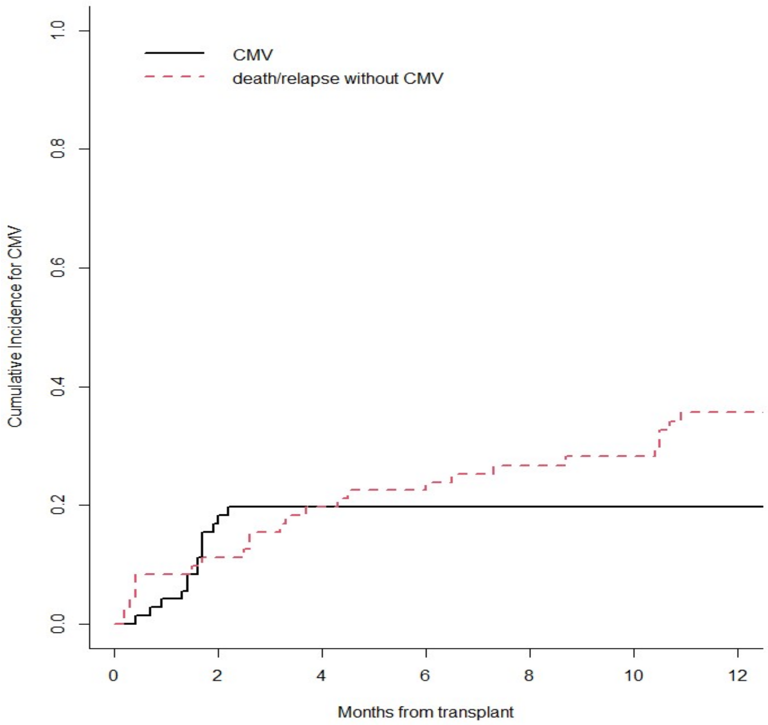

Figure S5. Cumulative incidence of clinically significant cytomegalovirus (CMV) reactivation.

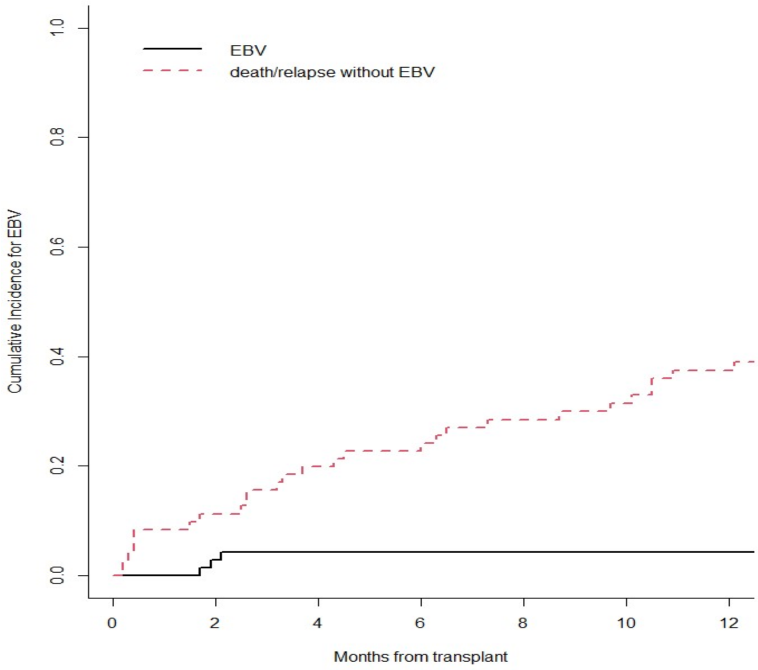

Figure S6. Cumulative incidence of and Ebstein-Barr (EBV) reactivation.

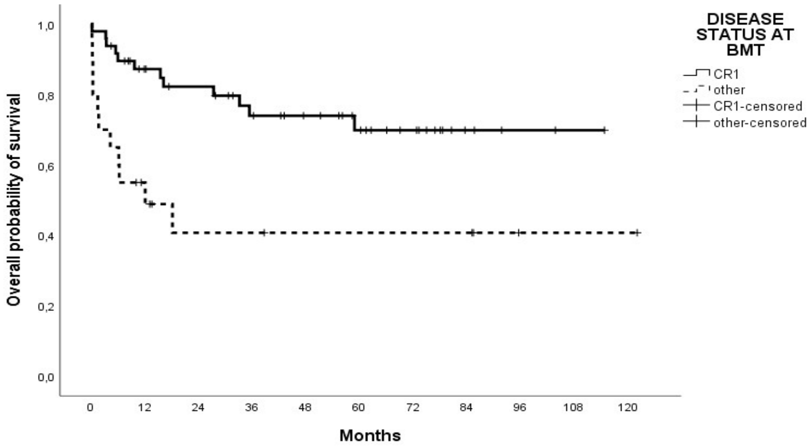

Figure S7. Overall survival considering disease status at transplantation

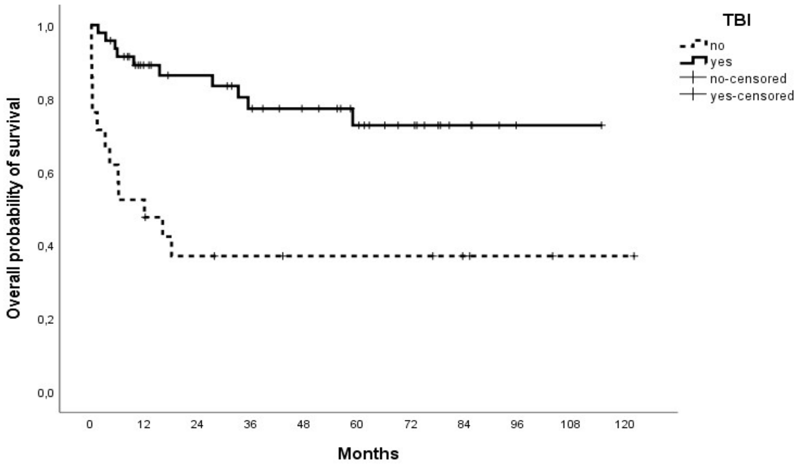

Figure S8. Overall survival: in detail TBI vs non-TBI
